# 3D-Printable Carbon Nanotubes-Based Composite for Flexible Piezoresistive Sensors

**DOI:** 10.3390/ma13235482

**Published:** 2020-12-01

**Authors:** Chaima Fekiri, Ho Chan Kim, In Hwan Lee

**Affiliations:** 1 Department of Precision Mechanical Engineering, Chungbuk National University, Cheongju 28644, Korea; cheimafekiri@chungbuk.ac.kr; 2 Department of Automotive Engineering, Andong National University, Andong 1375, Korea; hckim@andong.ac.kr; 3 School of Mechanical Engineering, Chungbuk National University, Cheongju 28644, Korea

**Keywords:** flexible tactile sensors, direct ink writing, flexible electronics, wearable technology, carbon nanotube nanocomposites, conductive polymers, printed electronics

## Abstract

The intersection between nanoscience and additive manufacturing technology has resulted in a new field of printable and flexible electronics. This interesting area of research tackles the challenges in the development of novel materials and fabrication techniques towards a wider range and improved design of flexible electronic devices. This work presents the fabrication of a cost-effective and facile flexible piezoresistive pressure sensor using a 3D-printable carbon nanotube-based nanocomposite. The carbon nanotubes used for the development of the material are multi-walled carbon nanotubes (MWCNT) dispersed in polydimethylsiloxane (PDMS) prepolymer. The sensor was fabricated using the direct ink writing (DIW) technique (also referred to as robocasting). The MWCNT-PDMS composite was directly printed onto the polydimethylsiloxane substrate. The sensor response was then examined based on the resistance change to the applied load. The sensor exhibited high sensitivity (6.3 Ω/kPa) over a wide range of applied pressure (up to 1132 kPa); the highest observed measurement range for MWCNT-PDMS composite in previous work was 40 kPa. The formulated MWCNT-PDMS composite was also printed into high-resolution 3-dimensional shapes which maintained their form even after heat treatment process. The possibility to use 3D printing in the fabrication of flexible sensors allows design freedom and flexibility, and structural complexity with wide applications in wearable or implantable electronics for sport, automotive and biomedical fields.

## 1. Introduction

Printed electronics, as the name implies, refers to the use of the additive manufacturing technology to create electronic components in a layer-by-layer printing method. The importance of printed electronics gained notice in the past few years both in the academic community and the electronic industry, as they offer a lot of advantages in regard to freedom of design, relatively fewer fabrication steps and scalability. They are also more environmentally friendly as opposed to subtractive lithography-based and patterning methods currently used for the fabrication of most electronics. Printed electronics aim to make electronic devices or circuits using printing technology instead of the much more expensive and complex electronics fabrication technology. For example, to fabricate a silicon-based integrated circuit (IC) chip, several hundreds of steps are required, from the preparation of a single crystal silicon substrate to making the components. This is an extremely complex process, including film deposition, lithography and acidic etching, not to mention a highly expensive process [1]. With the printing technology, the functional material can be directly printed with the desired patterns onto the substrate with only an additional curing process needed. As the electronics market keeps expanding at a high speed, especially with the widespread of the Internet of Things (IoT) with which the use of sensors is expected to reach the trillion by 2023 [2], printed electronics could offer a faster, cheaper and eco-friendlier way to produce electronic devices compared to the traditional manufacturing methods. This field gained tremendous attention in the past decade, largely due to the development and maturity of organic and inorganic nanomaterials (nanowires, nanotubes, nanoparticles,) which can be made into inks/pastes that can be then printed into patterns using different types of printing methods from roll-to-roll, to inkjet and extrusion processes [3,4,5,6,7,8]. The possibility of making polymers into conductive materials by doping certain molecules led to numerous studies working on the development and synthesis of functional flexible materials and nanocomposites using different types of fillers (metallic, ceramic, organic) [9,10]. For example, Wei et al. [11] showed the development of a 3D-printable graphene composite using a fused deposition modeling (FDM) process. Leigh et al. [12] formulated a conductive composite material suitable for 3D printing of sensors using Carbon Black fillers, where they used it to make a 3D printed “glove.” Nanocomposites that have high potential in flexible electronics are carbon nanotubes-based nanomaterials due to their low percolation threshold, mechanical flexibility for bending and stretching and high conductivity [13,14]. Nonetheless, there are still challenges on the way to improving the dispersion of the carbon nanotubes fillers within the binders to obtain easily printable inks with stable electrical properties—and in particular, for sensor applications, the stability of the sensing performance. For sensors with carbon-based nanocomposites, numerous works have been done to develop materials for 2D printing, such as screen printing [15], spray-coating, stamping and inkjet-printing [16]. More research is being conducted to develop materials using 3D printing fabrication methods. The works of Abshirini et al. [17,18] showed the 3D printing of a carbon-based nanocomposite for a highly stretchable strain sensor application where the sensor was tested under cyclic tensile loads for long-term performance, and the sensor was applied to monitor the bending of a human wrist. Emon et al. [19] developed a pressure sensor with 3D printed electrodes via a multi-material extrusion-based direct printing process. 3D printing in electronics applications can be used to fabricate the molds [20], the electrodes and sensing elements [21], the 3D printed substrates and the sensor body and fully-printed tactile sensors [22]. In the work by Guo et al. [23], a stretchable tactile sensor was 3D printed using a material extrusion process, and the sensor was tested for pulse monitoring and finger motions. With this variety of applications, different 3D printing technologies can be used. For instance, in the work of Vatani et al. [24] three different 3D printing processes (direct printing (DP), digital light processing (DLP), and projection stereolithography (PSL)) were investigated to fabricate tactile sensors. For printing conductive polymers, stereolithography (SLA), digital light processing (DLP) and direct ink writing (DIW) are the most in use. The direct ink writing technique, which is used in this work, is one of the promising extrusion-based processes for the deposition of carbon-based polymers and can also deposit a wide range of materials with different viscosities, including organic, inorganic and biomaterials. Such a system is usually not commercially available; therefore, in most works, it is developed in-house [25]. Another asset of this technique is its ability to produce complex shapes without the need for lithographic techniques, and the material is printed at room temperature, so it is suitable for heat-sensitive materials as well. It is also a scalable 3D printing technique where the resolution and sizes of the printed structures can be easily modified [26] by controlling the viscosity of the material, for example, [27], or the printing parameters.

In this work, we present a conductive 3D-printable composite based on carbon nanotubes that was extruded using DIW. The obtained material can be used in numerous applications in flexible electronics; here, a single-lined piezoresistive pressure sensor was fabricated; its sensing element was extruded via DIW. The sensor showed good sensitivity over a large pressure range and high flexibility without any remarkable degradation. The use of DIW technology also allowed the facile fabrication of complex flexible 3-dimensional shapes with high resolution and robust structures that did not collapse even when pressure was applied. This work also contributes to solving the problem of the printability of inks using solvents as dispersion media. Generally, the solvent affects the solubility of the ink, and thus its printability, which has been so far a challenging issue in this field, especially when printing multi-layer devices, as each printed layer can damage the underlying layer. This problem was treated here by also applying optimum printing parameters such as printing speed and extrusion pressure according to the rheological properties of the prepared ink.

## 2. Materials and Methods

### 2.1. Materials Formulation

**Figure 1:**
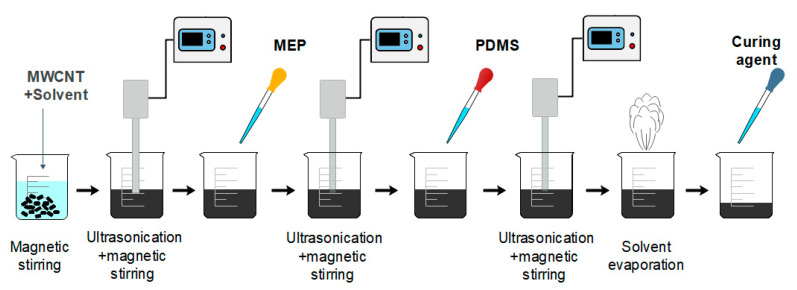
MWCNT-PDMS composite preparation process.

To make 8 wt % of MWCNT-PDMS composite, MWCNT (Industrial grade, NanoLab, Waltham, MA, USA), particles length of 5–20 μm, diameter 10–30 nm and purity >85%, were dispersed in a sufficient amount of IPA (>99%, Daejung Co., Ltd., Siheung, Korea), IPA/MWCNT weight ratio of 100:1 and the solution was sonicated for 30 min at 40 Hz. The sonicator (Q700, Qsonica L.L.C., Newtown, CT, USA) was operated at pulse mode with 60 s on and 20 s off to separate the agglomerated MWCNT particles due to the Van der Waals forces and to obtain a uniform dispersion inside the PDMS matrix without damaging the MWCNTs. Then 20 wt % of methyl group-terminated PDMS (MEP) (Sigma-Aldrich, St. Louis, MO, USA) was added and sonicated for 5 min. In the following steps, 80 wt % of PDMS prepolymer (Sylgard 184 Silicone Elastomer kit, Dow Corning, MI, USA) was added and the solution was ultrasonicated for an additional 5 min. The material was then left on a hot plate at a temperature that did not exceed 55 °C to evaporate the solvent. Upon completion, the curing agent for PDMS was added at a 10:1 ratio (PDMS/ curing agent weight ratio) and the entrapped bubbles remaining were eliminated with a vacuum desiccator. Figure 1 is a schematic representation of the composite material preparation process.

### 2.2. Sensor Fabrication

#### 2.2.1. Direct Ink Writing Process

**Figure 2:**
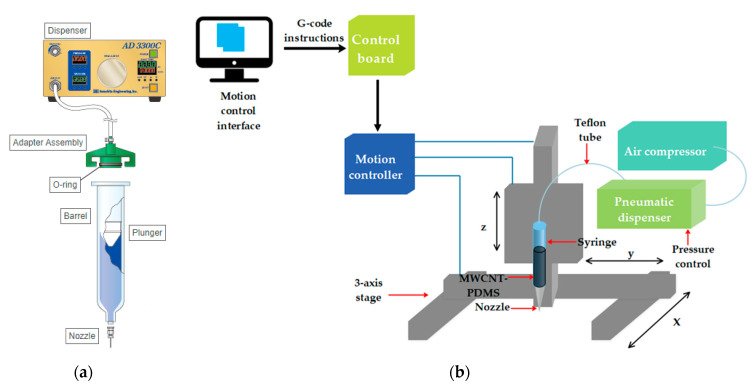
Schematic of the direct ink writing apparatus: (**a**) the pneumatic extrusion system; (**b**) overview of the DIW system with its different components.

The MWCNT-PDMS composite is printed using the DIW process. The DIW apparatus was built in house with a pneumatic extrusion system. The conductive polymer is extruded from a polypropylene barrel (PS10S, Iwashita Engineering Inc., Fukuoka, Japan) through a nozzle with a 0.51 mm inner diameter and 13 mm needle length (MN-21G-13, Iwashita Engineering Inc.). The barrel is connected to a pneumatic dispenser (ACCURA 8-DX, Iwashita Engineering Inc.) through a Teflon tube with 10 mm sized inner diameter and 1 m length (AA10n, Iwashita Engineering Inc.), and the compressed air is delivered with an air compressor (KDC-25, Keyang Inc., Seoul, Korea). The pneumatic extrusion system is presented in Figure 2a, the barrel is mounted on an x-y-z translation stage where the movements of each axis are controlled by a motion controller (Figure 2b). Each motion controller receives a G-code from a computer through a control board (LX504, Comizoa, Daejeon, Korea).

In the authors’ previous work, it has been shown that the line width and height of the printed specimens are correlated with the extrusion pressure and the printing speed and the stand-off distance [28]. For example, by increasing the printing speed, for a given dispensing pressure and given nozzle diameter, the line width and height will decrease, and vice versa. Controlling these parameters allows controlling the paste flow as well, which is another challenge regarding the DIW process, as the flow properties of the material depend on various factors, including the amount of residue solvent in the composite and the quality of dispersion.

**Figure 3:**
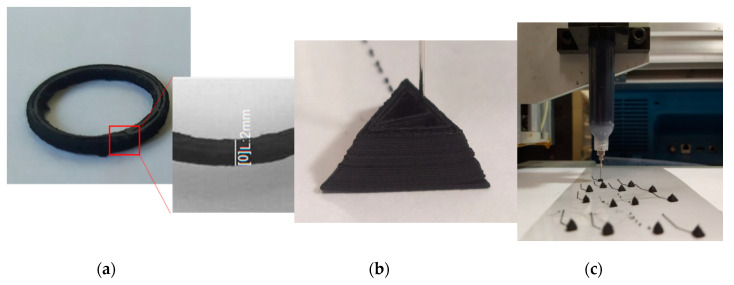
Fabricated 3-dimensional structures using DIW technology: (**a**) 3D ring of 10 layers of 200 µm single layer height and 1 mm width; (**b**) tetrahedron being printed: edge length = 15 mm with 75 layers. (**c**) Several smaller pyramid shapes being printed showing an easily scalable and repeatable printing process.

**Figure 4:**
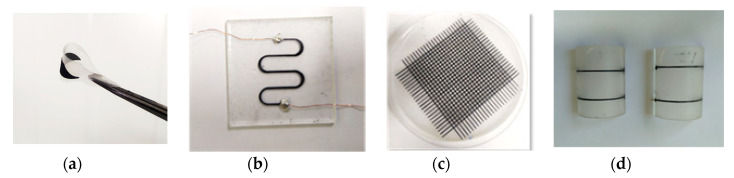
3D printed MWCNT-PDMS material patterns on soft substrates: (**a**) 3D printed film with extreme flexibility and bendability which shows that the sensor can be attached to non-conformal surfaces in practical applications; (**b**) 3D printed stretchable serpentine shape; (**c**) 3D printed grid forming 576 “taxels”; (**d**) printed MWCNT-PDMS composite on a non-conformal surface.

Figure 3 and Figure 4 present an example of a successful printing of multi-layered and single-layered high-resolution MWCNT-PDMS structures using the DIW technique.

Figure 3a shows a 3D printed ring with a 15 mm diameter and 2 mm height and 1 mm width. It was printed with a dispensing pressure of 200 kPa and a nozzle diameter of 510 µm. Feed rate was 5 mm/s and a single layer height is 200 µm. Figure 3b shows the printing progress of a tetrahedron of 15 mm height. To print this structure a nozzle with 260 µm was used. The dispensing pressure was 270 kPa, feed rate 3.3 mm/s and the single-layer height is 200 µm.

Figure 4 shows single-layered structures with different geometries that are extremely stretchable and bendable that can be attached to different surfaces.

Similar to a commercially available 3D printer, with this printing method the dimensions can be modified without modifying the structure, i.e., it is possible to fabricate the same geometrical shape with different sizes. Moreover, the fabrication process showed good repeatability where several 3-dimensional structures have similar geometrical features and qualitative accuracy.

#### 2.2.2. Sensor Fabrication

**Figure 5:**
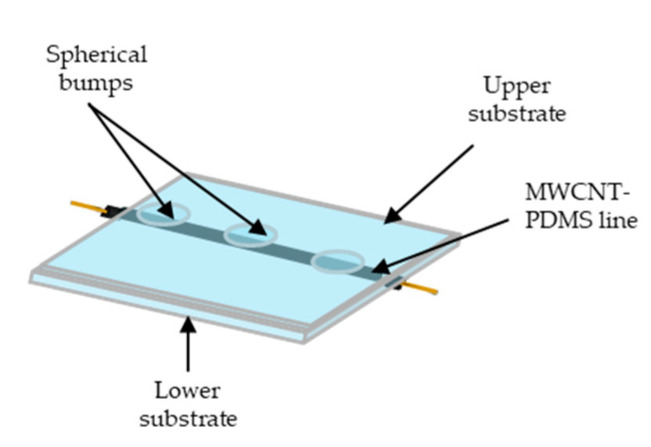
Illustration of the flexible pressure sensor.

The sensor has a simplified structure in order to focus on the study of the piezoresistive effect of the printed MWCNT-PDMS material under pressure. Therefore a single-lined sensor was fabricated (Figure 5). The printed line has a 2 mm width, 200 µm height and 40 mm length.

First, the PDMS elastomer and cross-linker were mixed and cast on 3D printed molds followed by an annealing process. The molds used for making the two PDMS substrates were printed using Digital Light Processing (DLP) process (IM2, Carima, Seoul, Korea) and made out of photopolymer resin (Carima GRN, CRM003, Carima) with dimensions of 40 mm × 40 mm × 1 mm.

The MWCNT-PDMS material is extruded onto the PDMS substrate with a nozzle diameter of 510 µm, an extrusion pressure of 250 kPa and a print speed of 5 mm/s. The line is then cured for 3 h at 70 °C and the second PDMS substrate was cured onto the layer with the printed material.

The upper substrate has 3 hemisphere bumps of 1 mm diameter which are creating contact points with the printed line to have an even pressure distribution at each point. The bump structure (also referred to in other works as porous structure, microdome structure, or force concentrator) has been applied in numerous studies [29,30,31,32]. The bump structure is a way to raise the sensitivity of the sensor as opposed to a flat layer. The bumps are bearing a higher concentration of pressure for the contact area.

Different sample sensors were fabricated under identical conditions and their geometrical features (height, length and width) were measured using a digital microscope (OSM-U, Dongwon Microscope, Seoul, Korea) to verify the repeatability of the printing process. For five samples, the standard deviation of the printed lines’ height was 0.023, for the lines’ width it was 0.032 and for the lines’ length 0.046. The low standard deviation shows minimal variation among the printed samples.

**Figure 6:**
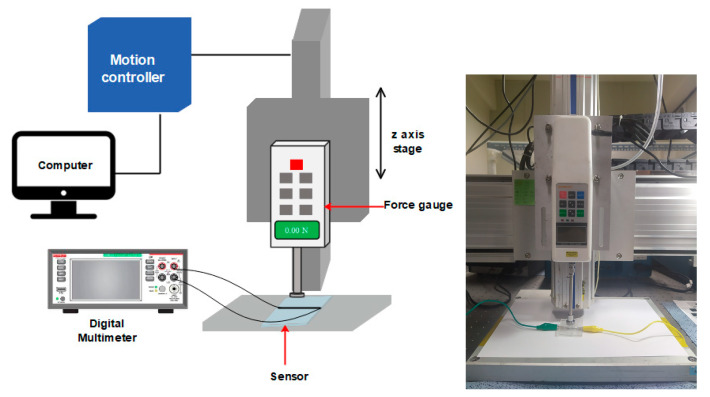
Custom build pressure measurement setup.

In order to test the performance of the sensor, a push–pull force gauge (SH-200, SUN DOO Instruments, Wenzhou, China) was used to apply a gradually increasing pressure and a digital multimeter (DMM6500, Keithley Instruments, Solon, OH, USA) was used to measure the resistance variation and to observe the response of the sensor to the amount of applied pressure. Figure 6 illustrates the testing setup. Without any initial load, the sensor has a non-zero value due to the initial resistance of MWCNT-PDMS material.

## 3. Results and Discussion

### 3.1. Chemicals and Materials

The dispersion of the MWCNT in the polymer matrix is still the subject of a lot of studies. The solvent is the vehicle that will allow the dispersion of the MWCNTs fillers in the polymer matrix. It should allow the ink to have good solubility, favorable viscosity and homogeneity. The choice of the solvent is therefore a crucial step, as it plays an important role in the overall performance of the sensor. For this work, several experiments using isopropyl alcohol (IPA) and toluene were conducted to find the optimum solvent and correspondent preparation process to obtain a 3D-printable MWCNT-PDMS nanocomposite. Although both solvents allowed good printability of the ink, some research [33,34] shows that IPA is considered a more suitable solvent for the stability of the dispersion of the MWCNT; the comparative study conducted by Ramalingame et al. [33] concluded that the IPA-based sensor “exhibits less hysteresis compared to that of THF based sensors,” and in the study by Kim et al. [34], the authors used IPA as a solvent medium to disperse the CNT particles in the polymer matrix, since “CNTs and PDMS are partially soluble in IPA.” Other works showed that when using toluene solvent the ink shows better sensitivity [35]. It is important to notice that in the above-mentioned studies, where IPA and toluene were used, the fabrication method was not a 3D printing-based method (mold casting in [35]).

Both solvents showed similar printability characteristics of the final composite, but for similar curing conditions, toluene showed better visual aspect, as IPA is more volatile compared to toluene, which creates micro-cracks if the printed structure is small and cured at high temperatures. On the other hand, IPA-based ink showed lower electrical resistance and better stability of the electrical properties; therefore, IPA was the chosen solvent for this work.

MWCNTs are a great choice for fillers when it comes to flexible conductive polymer-based composites because they have good mechanical properties, including high elastic moduli and tensile strength, excellent electrical conductivities, low percolation thresholds and high aspect ratios (length to diameter ratio) [36].

The polymer matrix used in the synthesis of the nanocomposite was polydimethylsiloxane (PDMS) (Sylgard 184 Silicone Elastomer kit, Dow Corning, MI, USA). It is a transparent and flexible polymer with good insulating and mechanical properties, without environmental toxicity, with biocompatibility and with simplicity of use. Compared to other flexible materials (PET, PI, PC, PMMA), PDMS has a low Young’s modulus. The specific Young’s modulus of PDMS is related to the ratio of the base and curing agent. To prepare the MWCNT-PDMS pressure-sensitive composite, a small amount of methyl group-terminated PDMS (MEP) was added to enhance the adhesion between MWCNT particles and PDMS matrix. As the MEP gets attached to the MWCNT particles, when introducing the PDMS to the MWCNT-IPA-MEP solution, the latter can make direct contact with the MEP phase surrounding the MWCNT tubes, which makes both PDMS and MWCNT-MEP homogenized and stable in the IPA solution [34].

### 3.2. Sensor Performance

**Figure 7:**
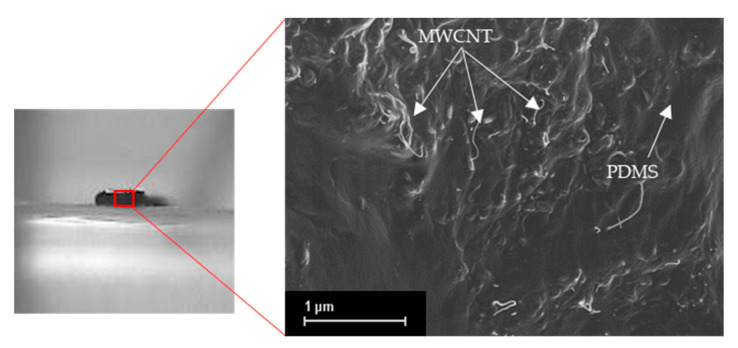
SEM image showing a cross-section of the MWCNT-PDMS composite where the dispersed MWCNT particles are forming a conducting network inside the PDMS matrix.

The MWCNT particles form conductive paths inside the matrix and create a network of resistors [37], and the distribution of the particles’ connections varies under external pressure, which leads to the resistance change. Figure 7 is a high magnification SEM image of a cross-section of the printed MWCNT-PDMS line with 0.5 mm height, showing the dispersion of MWCNT particles in the PDMS matrix. It is believed that during compression, the conductive networks undergo a process of destruction and formation of the percolation channels of the particles’ network. The deformation of the CNT-polymer nanocomposite perpendicular to the uniaxial pressure causes alterations in the conductive paths [38]. In this case, the electrical resistivity of the material can either increase or decrease. This property is related to the aspect ratio of the filler particles. Fillers with a low aspect ratio such as carbon blacks showed a decrease of the electrical load under external pressure, this effect is called the negative pressure coefficient of resistance (NPCR) effect. On the other hand, the resistance increases under pressure with high aspect ratio particles such as carbon nanotubes, and in this case, it is a positive pressure coefficient of resistance (PPCR) effect [39].

**Figure 8:**
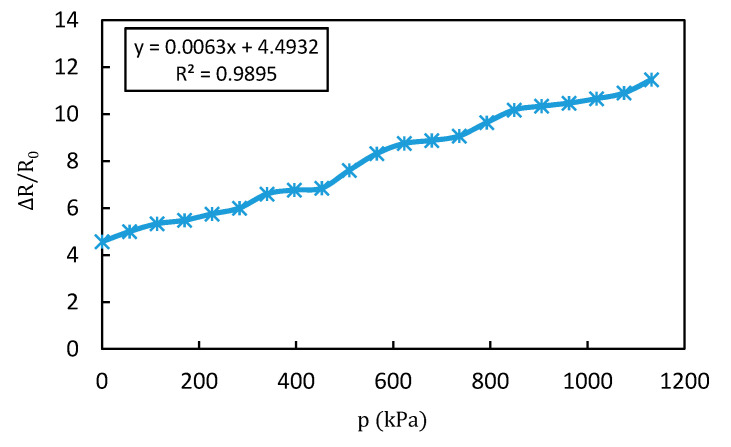
The sensor performance over a large range of applied pressure.

**Figure 9:**
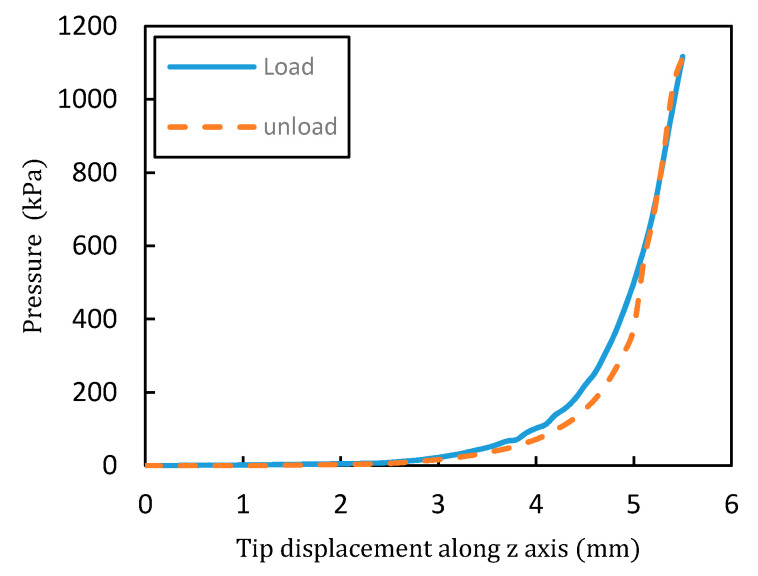
Applied pressure according to the displacement of the push–pull gauge tip (along the z-axis).

A progressive pressure was applied gradually to the sensor and the result of the resistance variation according to the applied pressure is shown in Figure 8. The pressure was applied by placing the sensor on the optical table and moving the push–pull gauge according to the z-axis which is perpendicular to the sensor plane. The movement of the gauge was incremented by 0.1 mm from top to bottom until reaching its maximum capacity. Figure 9 shows the relationship between the applied pressure and the displacement of the push–pull gauge. The tip of the push–pull gauge has a diameter of 15 mm and the pressure measurement range was from 0 to 1123 kPa.

It is shown in Figure 8 that the resistance increases with the increased pressure; the sensor then exhibits a positive pressure coefficient of resistance (PPCR) effect. The sensor shows a linear response over a large range of applied pressure from 0 to 1132 kPa.

According to the graph in Figure 8, the output resistance can be expressed as a function of pressure (p) according to Equation (1).

(1) ΔR (p)= 6.3p+4.49, p ϵ [0, 1132 kPa]

where ∆R (p) is the output resistance change and p is the applied pressure.

### 3.3. Sensitivity

By definition, the sensitivity is the ratio of the small increment of the output (∆R) to the small increment of the input stimulus (p), given by the following formula:

(2) S=|ΔRR0p|

∆R is the variation of the output resistance of the sensor, $R_{0}$ is the initial resistance and p is the applied pressure. The sensitivity of the linear measurement system is a constant and can be obtained from the slope of the static characteristic curve. The sensitivity of the sensor is 6.3 Ω/kPa.

### 3.4. Hysteresis Error

A hysteresis error is a deviation of the sensor output at a specified point of the input signal when it is approached from the opposite directions which can be expressed as a percentage of the full scale (%FS)

(3) $$H_{e}=\frac{\left|\Delta H_{e}{}_{\max}\right|}{\mathrm{FS}}\;\times 100\%$$

**Figure 10:**
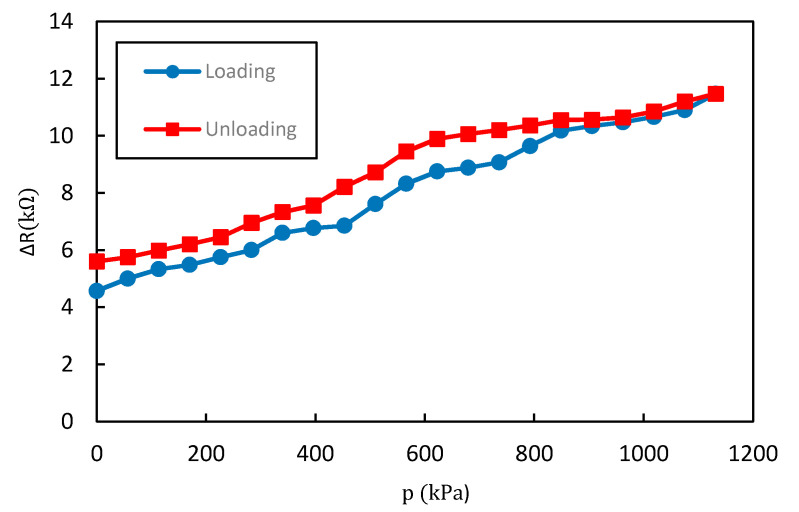
Resistance change relative to the loading and unloading of pressure P.

$H_{e}$ is hysteresis error; $\Delta H_{e}{}_{\max}$ is the maximum deviation between the load phase and the unload phase; FS is the full-scale output reading. Figure 10 is a representation of the hysteresis of the sensor. For an overall range of 1132 kPa, the sensor showed 20.24% of hysteresis error throughout its working pressure range.

Given the wide range of applied pressure for which the sensor was tested, it can be considered that the sensor showed low hysteresis. The cause of this hysteresis phenomenon is the energy dissipation due to the viscoelastic property of the elastomer. Significant viscoelastic behavior of the PDMS layer can limit the performance of the pressure sensor in terms of relaxation time and response. Nevertheless, our sensor showed low hysteresis due to the structure of the upper PDMS layer. The hemispherical bumps create space between the two substrates of the sensor which can reduce the viscoelastic behavior of the PDMS compared to a flat structure, leading to a short relaxation time under external pressure.

### 3.5. Linearity Error

The linearity error (often referred to as the nonlinearity error) is specific for sensors for which the transfer function may be approximated by a straight line [40]. It is usually expressed in percentage of span, as given by Equation (4).

(4) $$\delta_{\mathrm{NL}}=\frac{\Delta L_{\max}}{\mathrm{FS}}\times 100\%$$

where $\delta_{\mathrm{NL}}$ is the nonlinearity error (in % of the full scale); $\Delta L_{\max}$ is the maximum deviation between the real transfer function and the approximation straight line.

There are several ways to specify linearity depending on how the line is superimposed on the transfer function (“terminal points” method or “best straight-line” method) [40]. In this work, we used the least square fit method. Therefore, the nonlinearity value presented refers to the least-squares linearity.

From Figure 8 and Equation (4), we can determine that the value of the nonlinearity error is 4.35%. This is a low nonlinearity error over a wide measurement range.

### 3.6. Dynamic Performance

**Figure 11:**
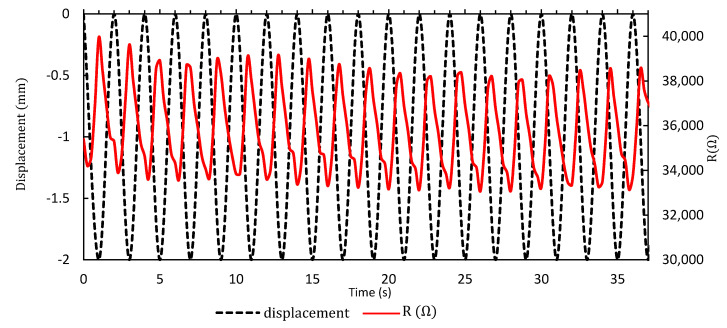
Dynamic response of the sensor according to applied pressure. The variation is shown according to the displacement of the tip of the push–pull gauge where the position of −2 mm corresponds to the loading state and the position of 0 mm corresponds to the unloading state.

For the dynamic response of the sensor, cyclic loading and unloading of the pressure were applied to the sensor, and the electrical resistance variation was measured in real-time. The loading frequency was 0.5 Hz and the pressure variation spanned 0–14 kPa. The dynamic characteristic of the sensor is shown in Figure 11. The graph is showing that the response to an external load was immediate and no noticeable relaxation time was observed for the loading and unloading on the pressure sensor. The frequency of the response signal of the sensor was also 0.5 Hz.

## 4. Conclusions

3D printing technologies offer a lot of advantages in regard to freedom of design, relatively fewer fabrication steps, versatility and more environmental friendliness, as opposed to subtractive lithography-based and patterning methods currently used for the fabrication of most electronics; therefore, it is important to adapt 3D printing to the fabrication of flexible sensors. In this work, a high-sensitivity piezoresistive sensor was developed using the DIW technique to deposit the MWCNT-PDMS composite onto a flexible PDMS substrate. The MWCNT-PDMS was formulated for 3D printing using an extrusion-based method. The DIW system was also developed in order to print functional materials with different viscosities, and we were able to print multi-layered 3-dimensional structures with high resolution. The material showed good printability and the same geometrical features after the curing process. The fabricated sample sensor with single line extruded MWCNT-PDMS material showed good performance over a wide range of measurements: high sensitivity, relatively low hysteresis error and low non-linearity error; and an immediate response to the loading and loading cycles. Giving the fact that 3D-printable pressure-sensitive materials for sensor applications are part of a field which is still in its infancy presenting a lot of challenges, this work is a contribution which could open up new possibilities for research and applications to further optimize the material development for different applications, including wearable electronics, prosthetics and robotics applications.
